# Preparation and Performance Evaluation of Castor Oil-Based Asphalt Regeneration Agent

**DOI:** 10.3390/ma17092078

**Published:** 2024-04-28

**Authors:** Pan Pan, Yibo Chen, Xinhe Hu, Bingquan Dai, Xiaodi Hu, Ning Wang

**Affiliations:** 1 School of Civil Engineering and Architecture, Wuhan Institute of Technology, Wuhan 430205, China; panp@wit.edu.cn (P.P.);; 2 Hubei Provincial Engineering Research Center for Green Civil Engineering Materials and Structures, Wuhan 430073, China; 3 The College of Post and Telecommunication of Wuhan Institute of Technology, Wuhan 430073, China; 4 China Three Gorges Construction Engineering Corporation, Chengdu 610095, China

**Keywords:** regeneration agent, castor oil, laboratory aging, asphalt binder, RAP asphalt mixture

## Abstract

Regeneration agents play a critical role in modifying the mechanical properties and durability of RAP asphalt mixtures. This paper aimed to develop a castor oil-based asphalt regeneration agent. The effects of this regeneration agent on the pavement performance of laboratory-aged asphalt and an RAP asphalt mixture were comparatively studied by a series of laboratory tests. For the developed castor oil-based asphalt regeneration agent, the weight ratio of the castor oil to dibutyl phthalate was determined as 1:4. Moreover, the regeneration effectiveness of the castor oil-based regeneration agent was tested on three laboratory-aged asphalt binders and an RAP asphalt binder; the penetration, softening point and ductility of the RAP asphalt binder recovered to 83 dmm, 50.3 °C, and more than 100 cm, respectively. The optimum content of the regeneration agent was 5% by the weight of the aged asphalt binder. Furthermore, the castor oil-based regeneration agent could effectively restore the pavement performance of an RAP asphalt mixture. In this study, the RAP percentage can reach up to 60% by the weight of the HMA mixture using the castor oil-based asphalt regeneration agent according to the Chinese specification.

## 1. Introduction

The maintenance of asphalt pavement not only produces a large amount of asphalt waste, but also consumes a considerable quantity of asphalt binder and mineral aggregate [1,2,3]. In China, the amount of RAP (reclaimed asphalt pavement) is very huge and tends to increase annually [4,5]. Undoubtedly, application of the RAP in new asphalt mixtures can reduce the accumulation of RAP material and the demand for the non-renewable pavement materials, e.g., mineral aggregate and asphalt binder [6,7]. It has been shown that the material cost would be reduced by 14~34% when the content of RAP ranges from 20% to 50% by the total weight of asphalt mixture [8]. The Ministry of Transport of China proposed that the RAP utilization rate should be over 80% by 2025, aiming to promote green transportation development [9]. Therefore, recycling the RAP for the maintenance or construction of asphalt pavement is highly significant from the policy perspective.

According to the construction temperature, there are three methods for recycling the RAP, including hot recycling, cold recycling, and warm recycling technologies [10,11]. There are many factors affecting the pavement performance of a recycled asphalt mixture, e.g., the new asphalt, aggregate gradation, the regeneration agent, the aged asphalt of the RAP, the particle characteristics of the RAP, the amount of RAP material, and the production and compaction temperatures during the construction stage [12,13,14]. Considering both the pavement performance and durability, the RAP percentages range from 20% to 30% by the weight of the asphalt mixture, and it is commonly used in the middle layer or bottom layer of asphalt pavement in actual pavement engineering. Hence, it has become a hot topic to increase the RAP percentage in asphalt mixtures without sacrificing the pavement performance.

In recent decades, a regeneration agent is usually used to recover the aged asphalt binder and improve the bonding performance between the RAP particles and the new aggregate particles to enhance the mechanical properties and durability of an RAP asphalt mixture [15]. The most common asphalt regeneration agent is a mineral oil-based regeneration agent according to the base material source. In addition, different types of additives are commonly added and mixed with the base material to modify the regeneration performance of the regeneration agent [16,17]. However, mineral oil is non-renewable and its production process results in environmental pollution [18]. Therefore, some researchers all over the world have prepared asphalt regeneration agents with vegetable oil as the base material.

In their published papers, the regeneration effects of different vegetable oil-based and mineral oil-based regeneration agents on aged asphalt were compared, and the results showed that the vegetable oil outperforms the mineral oil-based regeneration agents [19,20,21]. Zheng et al. studied the regeneration effect of vegetable oil from the aspects of permeability and molecular level. They found that vegetable oil shows better compatibility with the aged asphalt binder, owing to its greater permeability and stronger molecular activity [22]. For the same aged asphalt binder, the amount of vegetable oil was only one-third that of the mineral oil-based regeneration agent to obtain the same regeneration effect [23]. Since plant resources are abundant and renewable, it is significant to develop the vegetable oil-based regeneration agent.

In addition, a few studies were conducted on the regeneration properties of fresh vegetable oil [24,25]. Zhong found that the “amphipathic structure” in most vegetable oils has excellent dissolution and dispersion ability for asphaltene. Among them, castor oil was considered as one of the most promising vegetable oil-based materials to prepare regeneration agents due to its good thermal stability, storage stability, and regeneration effect. However, a low dosage of castor oil cannot obtain a satisfactory regeneration effect while a high dosage might increase the risk of moisture damage and permanent deformation of the RAP pavement [26,27]. Moreover, the content of RAP in an asphalt mixture is limited in practical engineering to below 30% by the weight of the mixture. Therefore, a modified castor oil-based regeneration agent is needed for improving the RAP percentage without sacrificing the pavement performance of an asphalt mixture.

**Figure 1:**
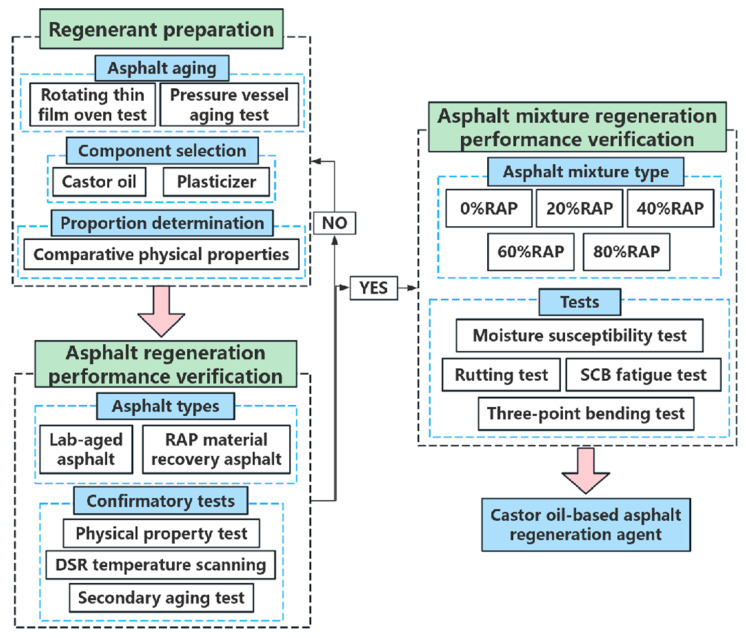
A technical diagram of this study.

This paper aimed to develop a new castor oil-based asphalt regeneration agent. The effects of the regeneration agent on the pavement performance of laboratory-aged asphalt and an RAP asphalt mixture were comparatively studied. Figure 1 shows a technical diagram of the present study.

## 2. Materials and Methods

### 2.1. Raw Materials

In this paper, all the raw materials came from Wuhan, China. Three kinds of 70# paving asphalt were selected, including JL AH-70, SL AH-70, and Shell AH-70. Table 1 shows their physical properties.

The gneiss aggregate had a density of 2.649 g/cm^3^ and a particle size less than 26.5 mm. The mineral filler was limestone filler with a density of 2.745 g/cm^3^. Their physical and mechanical properties met the requirements of the Chinese specification JTG F40. The reclaimed asphalt pavement (RAP) material, obtained from a pavement project in Macheng City, Hubei, had an asphalt content of 4.6% and a particle size less than 19 mm.

The raw materials of the asphalt regeneration agent included castor oil, Plasticizer ESO, Plasticizer TC, and Plasticizer DP. Since all the raw materials are liquid at 25 °C, during the mixing of the raw materials and the application of the regeneration agent, a low-speed shear device was used to shear at a speed of 500 r/min for 15 min. Table 2 shows the performance of the raw materials for preparing the asphalt regeneration agent.

### 2.2. Sample Preparation

#### 2.2.1. Preparation of Aged Asphalt

The aged samples were prepared according to the Chinese specification of JTG E20 [28]. Firstly, the short-term-aged asphalt binders were obtained by a rotating thin-film oven test (RTFOT, JTG E20 T0610) at 163 °C for 85 min. Then, the short-term-aged asphalt binders were subjected to the pressure vessel aging tests (PAV, JTG E20 T0630) at 100 °C for 20 h under 2.1 MPa to obtain the long-term-aged asphalt binders.

In addition, actual aged asphalt binder was extracted from RAP to verify the regeneration performance of the castor oil-based regeneration agent. Firstly, the RAP mixtures were cleaned by an extractor to obtain a trichloroethene bitumen concentrate. Then, the mixed solution was centrifuged at a speed of 3000 r/min for 5 min to remove the sediment. The solution after centrifugation was subjected to the rotary evaporation test according to JTG E20 T0727 [28]. Table 3 shows the physical properties of the aged asphalt binders.

#### 2.2.2. Asphalt Mixture Design

**Figure 2:**
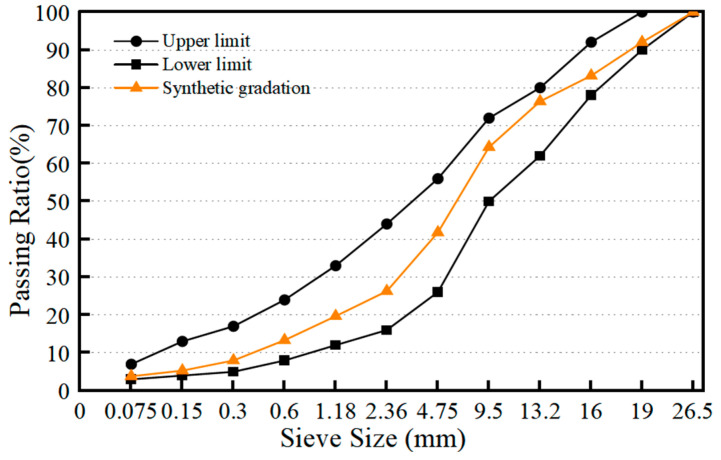
Chart of aggregate gradation.

Figure 2 shows the gradation curve of the asphalt mixture with the nominal maximum size of 19 mm. The upper and lower limits of gradation followed the Chinese specification of JTG F40-2004 [29]. The content of the mineral filler was 4% by weight of the aggregate and the optimum asphalt content was 4.4% by weight of the asphalt mixture.

### 2.3. Test Method

#### 2.3.1. Physical Properties Test of Asphalt

The physical properties of the asphalt binder including penetration (25 °C), ductility (15 °C), and softening point were studied in accordance with Chinese specifications JTG E20 T0604, T0605, and T0606, respectively [28].

#### 2.3.2. DSR Temperature Scanning Test of Asphalt

The DSR temperature scanning test was carried out at high temperature (40~80 °C) and at low temperature (0~40 °C) with a temperature interval of 5 °C. The cracking factor (G*cos^2^δ/sinδ) and rutting factor (G*/sinδ) of the asphalt were calculated by using the complex shear modulus G* and phase angle δ.

#### 2.3.3. Pavement Performance Test of Asphalt Mixture

According to the Chinese specification JTG E20, the high-temperature performance and the low-temperature performance of the asphalt mixtures were evaluated by wheel tracking test (T0719) and the three-point bending test (T0715), while the moisture susceptibility of the asphalt mixtures was determined by the Marshall stability test (T0709) and the freeze–thaw split test (T0729).

#### 2.3.4. SCB Fatigue Test

The SCB fatigue test was carried out to evaluate the fatigue performance of the aged and rejuvenated asphalt mixtures. The stress control model was adopted and the loading pressure of each asphalt mixture was 0.7 times its maximum load. Four test repetitions were prepared for each mixture and the average value was used. The frequency of cyclic loading was 10 Hz and the test temperature was 25 °C. The loading cycles were defined as fatigue life when the samples was damaged.

## 3. Preparation of Asphalt Regeneration Agent

### 3.1. Selection of Asphalt Regeneration Agent Materials

#### 3.1.1. Effect of Castor Oil on Physical Properties of Asphalt Binder

**Figure 3:**
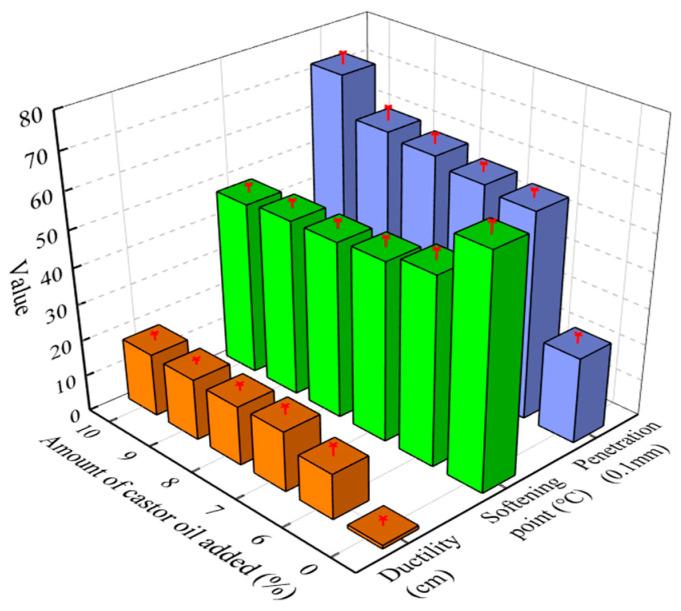
Physical properties of aged asphalt mixed with castor oil.

Castor oil was adopted as the base material for preparing the asphalt regeneration agent. Castor oil with five different dosages (6%, 7%, 8%, 9%, 10%) was added to the long-term-aged JL AH-70. Figure 3 illustrates the effects of the aging process and the castor oil on the physical properties of the asphalt binders. The penetration increased with the increase in castor oil content. When the castor oil content increased from 0% to 10%, the penetration of the aged asphalt binder increased from 24 dmm to 72 dmm. Since penetration is related to the stiffness of asphalt, asphalt binder with low penetration has high stiffness. The result implied that castor oil can efficiently soften and recover aged asphalt binder.

Since asphalt binder would become harder after aging and the softening point increase due to the aging effect, it can be assumed that an aged asphalt mixture is less prone to permanent deformation at high temperature. The softening point showed a clear decline trend with the castor oil. The softening point of aged asphalt with 10% castor oil was reduced by 25% compared with the original asphalt. This indicated that castor oil softened the aged asphalt as well, which was in accordance with the penetration results.

The test results for the physical properties confirmed that castor oil can restore the performance of asphalt binder. The reason might be that it contains a large number of aromatic components and increases the content of light components in asphalt binders. Compared with the penetration and softening point, the improvement of ductility was not significant. When the castor oil content was 10%, the ductility of aged asphalt was only 17 cm and could not meet the requirement of the JTG E40-2004 specification in China.

Ductility is generally used to evaluate the low-temperature cracking performance of asphalt binder. Asphalt binder with low ductility would increase the risk of the cracking of an asphalt pavement at low temperature. Therefore, plasticizers were adopted as a modifier to improve the low-temperature performance of the regenerated asphalt binder. The penetration and softening point of the aged asphalt binder with 8% castor oil was similar to the original asphalt. On this basis, the effect of plasticizers on the ductility of aged asphalt binders was investigated in follow-up research.

#### 3.1.2. Effect of Plasticizers on Physical Properties of Asphalt Binder

**Figure 4:**
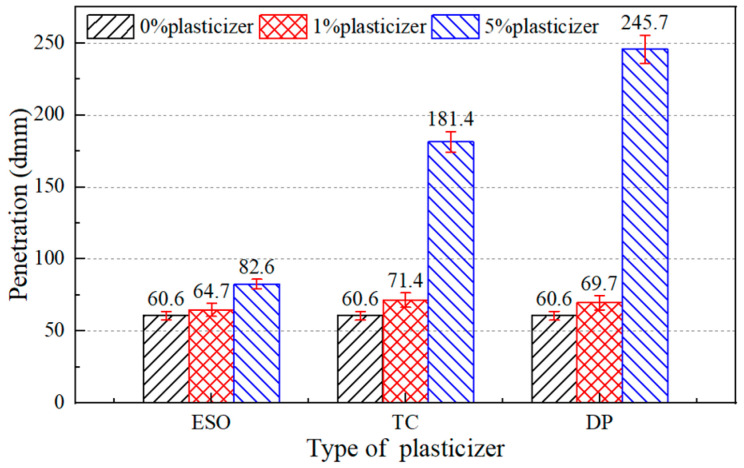
Penetration of 8% castor oil and plasticizer asphalt binders.

**Figure 5:**
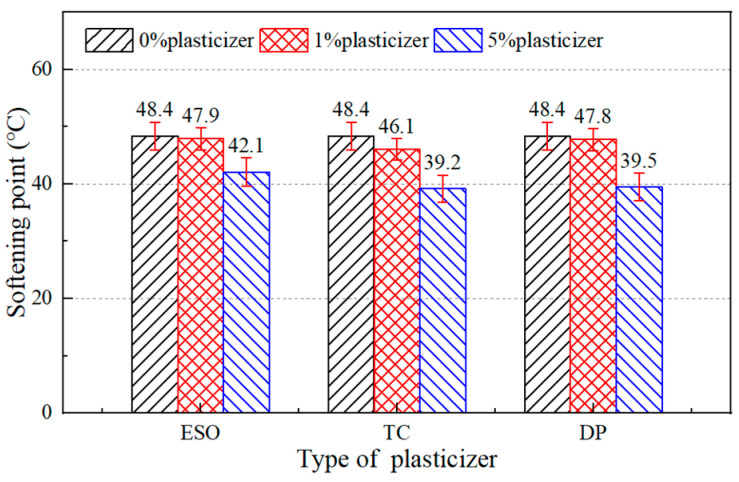
Softening point of 8% castor oil and plasticizer asphalt binders.

**Figure 6:**
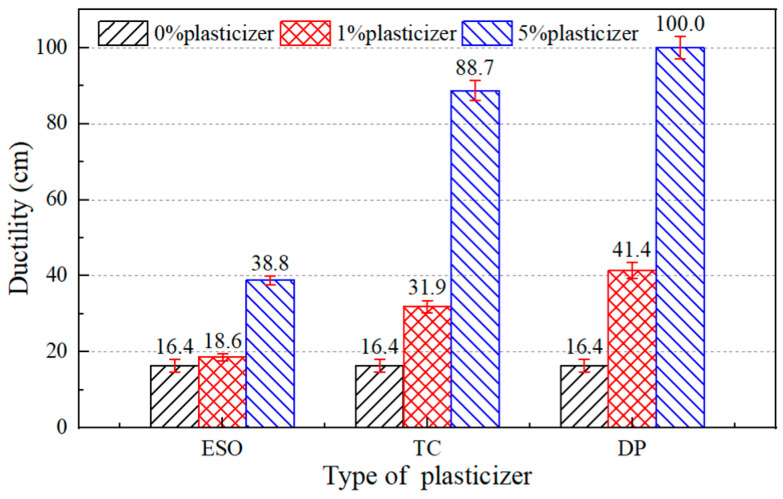
Ductility of 8% castor oil and plasticizer asphalt binders.

Three common plasticizers, including epoxy soybean oil (ESO), tributyl citrate (TC), and dibutyl phthalate (DP), were used in this study. On the basis of adding 8% castor oil into the asphalt binder, plasticizers with 5% (high content) and 1% (low content) by the weight of the asphalt binder were added as well. The purpose of the low content was to find out whether the plasticizer can function effectively at lower content; the purpose of the high content was to find out whether the plasticizer would have saturation effect or performance degradation at higher content. The physical properties of the asphalt binders were tested to compare the effects of the different plasticizers on the performance of aged asphalt binders. At the same time, the approximate range of plasticizer content which can restore the ductility to the standard value was estimated. The test results are shown in Figure 4, Figure 5 and Figure 6. Since castor oil showed good recovery effects on the penetration and softening point of aged asphalt binders, more attention was paid to the effect of plasticizers on improving the ductility.

As shown in Figure 6, the ductility improvement of aged asphalt binders with dibutyl phthalate was greater than for those with tributyl citrate and epoxy soybean oil. Of all the test results, only aged asphalt binder containing 5% dibutyl phthalate met the ductility requirements of the Chinese specification. It could be concluded that dibutyl phthalate showed the best recovery effect on ductility and thus it was selected as another component of the asphalt regeneration agent.

Moreover, the penetration of aged asphalt binders increased and the softening point decreased with the addition of plasticizers. After adding 5% dibutyl phthalate, the softening point and penetration were 39.5 °C and 246 dmm, respectively. Too low a softening point might cause the risk of permanent deformation of the pavement. For instance, the softening point of asphalt binder is required to be higher than 46 °C in China. Therefore, it was necessary to determine the optimum ratio of castor oil and plasticizer for obtaining a regeneration asphalt mixture with sufficient stability to avoid cracking and permanent deformation.

### 3.2. Asphalt Regeneration Agent Proportion Design

**Figure 7:**
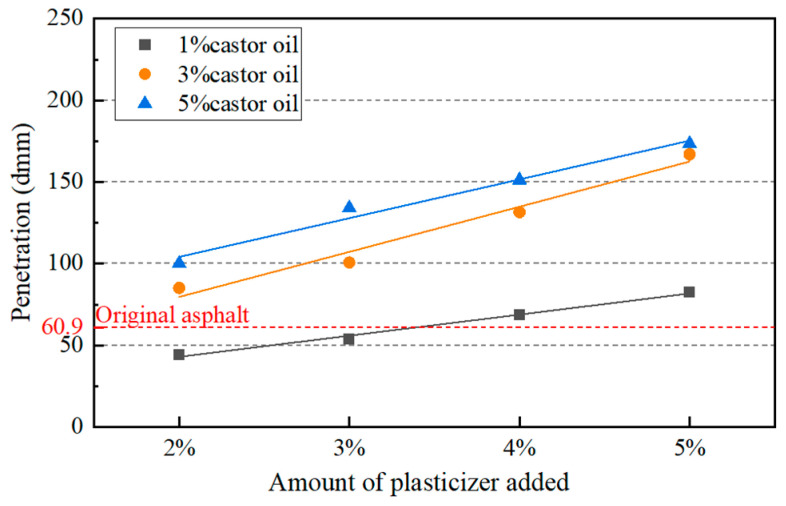
Penetration of aged asphalt with different amounts of additives.

**Figure 8:**
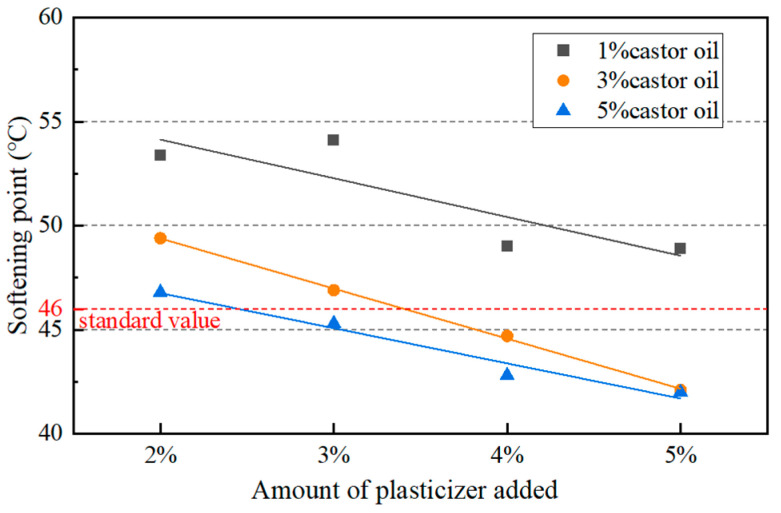
Softening point of aged asphalt with different amounts of additives.

**Figure 9:**
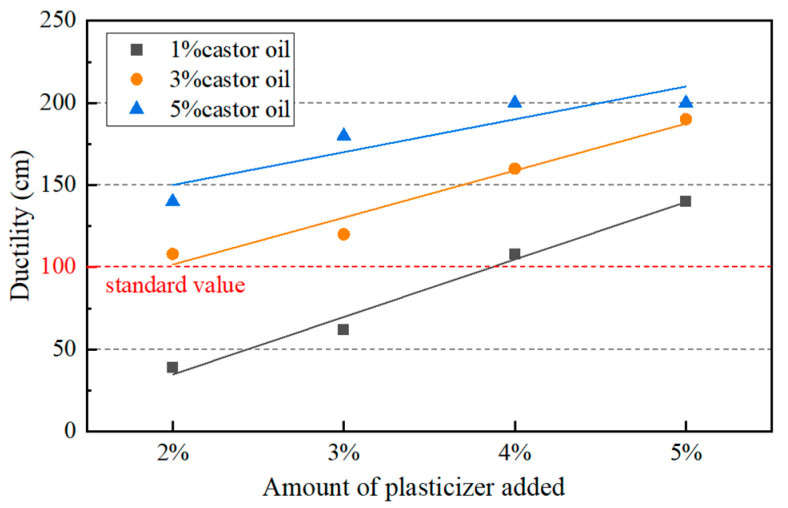
Ductility of aged asphalt with different amounts of additives.

Under the combined effects of castor oil and plasticizer, the penetration of aged asphalt binder with 8% castor oil and 5% plasticizer was too high and the softening point could not meet the Chinese standard. Since the castor oil has a significant softening effect on the aged asphalt binder, the content of castor oil was decreased, which ranged from 1% to 5% in further study. In addition, the content of plasticizer ranged from 2% to 5%. The physical properties of asphalt binders were tested for all combinations of the two additives with different contents. The test results and their comparison with the Chinese specification are shown in Figure 7, Figure 8 and Figure 9.

The softening point and ductility needed to be more than 46 °C and 100 cm according to the specification, respectively. So, only five combinations of dosages met the requirements. Considering both the physical properties and the cost, it was determined that the ratio of castor oil to dibutyl phthalate was 1:4 and the amount of asphalt regeneration agent was 5% of asphalt. The penetration and the softening point of the asphalt were 69 dmm and 48.9 °C, respectively, and the ductility was more than 100 cm. In order to avoid accidental phenomena, the castor oil-based asphalt regeneration agent was added to different kinds of aged asphalt binders and asphalt mixtures to verify its regeneration performance.

### 3.3. Validation of Regeneration Performance

#### 3.3.1. Effect on Physical Properties of Aged Asphalt Binder

**Figure 10:**
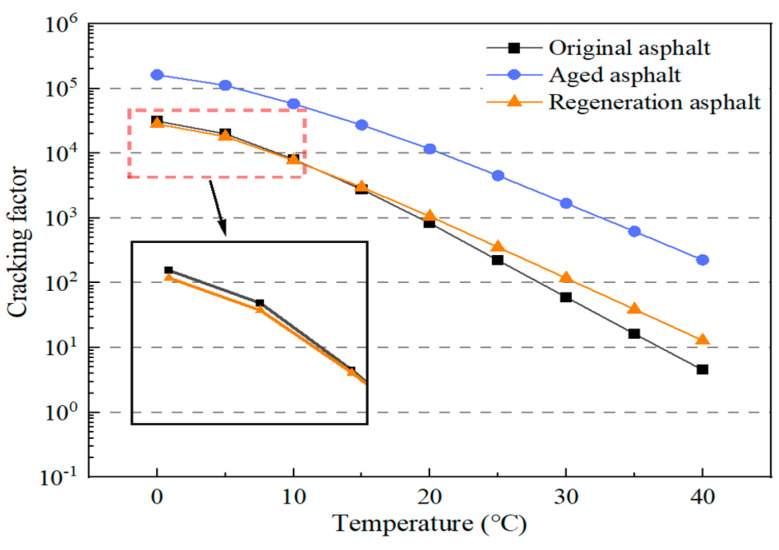
Low-temperature scanning of asphalt DSR test.

**Figure 11:**
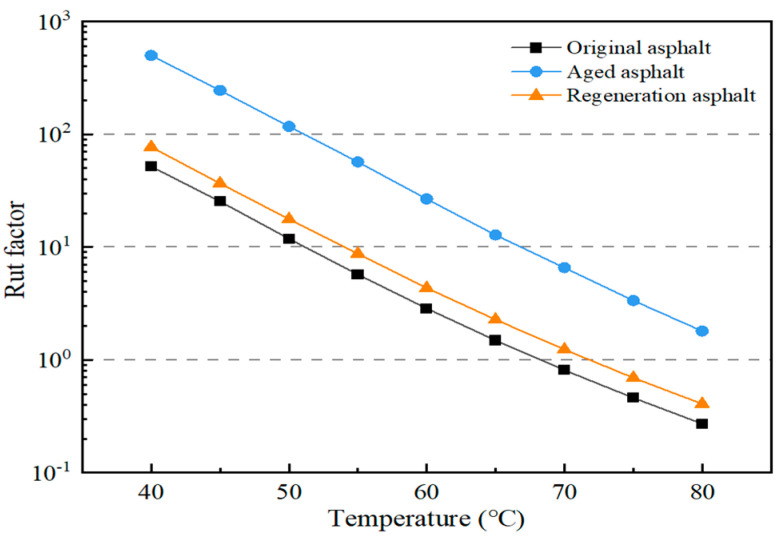
High-temperature scanning of asphalt DSR test.

DSR temperature scanning test results of the original asphalt, the aged asphalt, and the regeneration asphalt are shown in Figure 10 and Figure 11. A greater cracking factor means worse low-temperature performance while a greater rutting factor means better high-temperature performance. The high-temperature performance was improved and the low-temperature anti-cracking performance was degraded after aging. The DSR temperature scanning test results of the regeneration asphalt were similar to those of the original asphalt. The performance of the aged asphalt could be restored to the original asphalt level after adding the regeneration agent. This implied that the castor oil-based agent has good regeneration performance.

**Figure 12:**
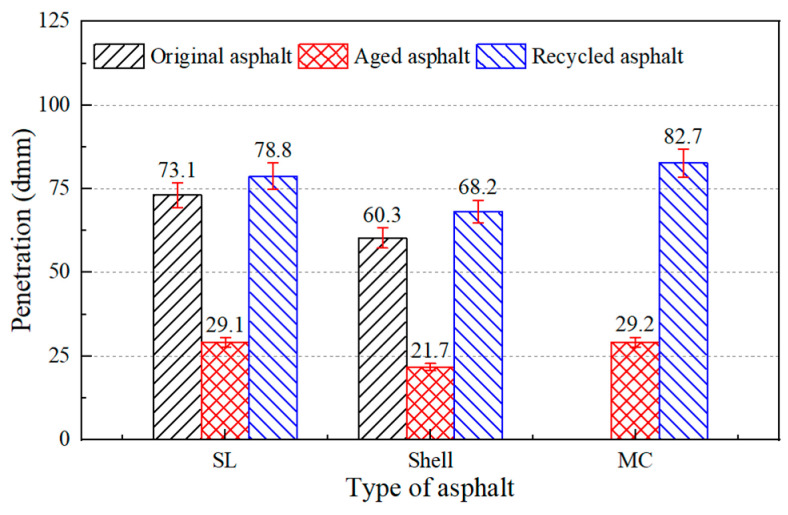
Penetration of aged asphalt and asphalt with regeneration agent.

**Figure 13:**
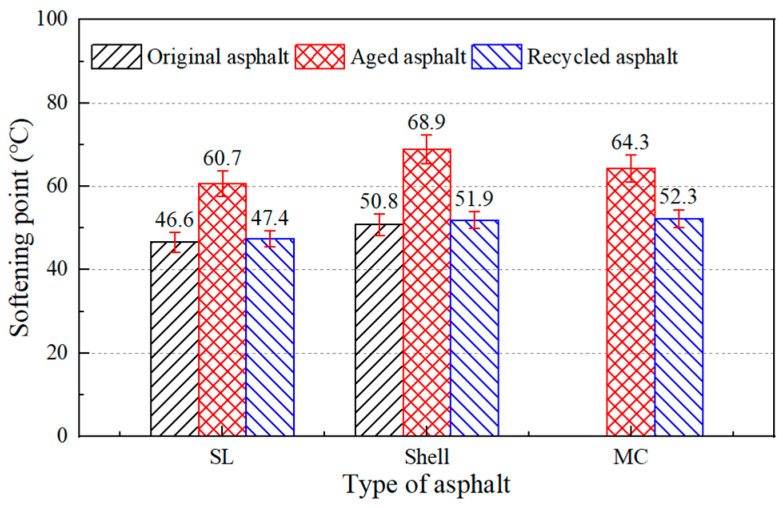
Softening point of aged asphalt and asphalt with regeneration agent.

**Figure 14:**
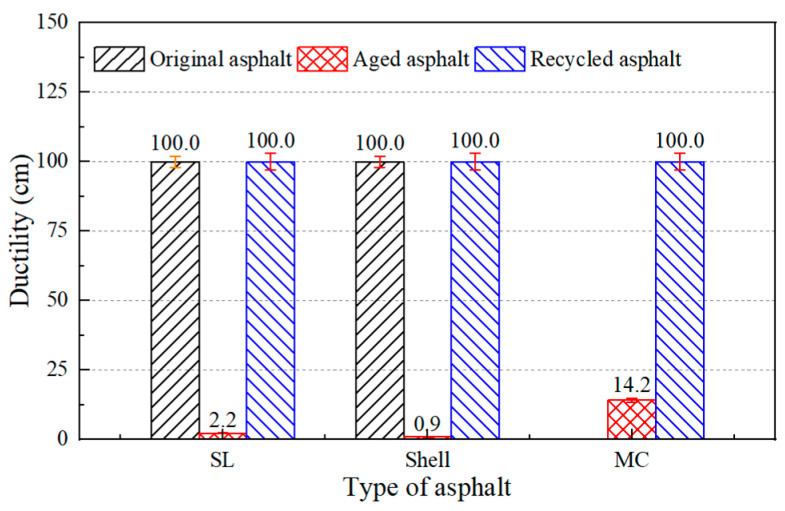
Ductility of aged asphalt and asphalt with regeneration agent.

In addition, there were three different asphalt binders to test the regeneration effect of the developed regeneration agent. The SL and Shell aged asphalt binders were obtained by laboratory aging tests and the actual aged asphalt (MC) was reclaimed from RAP. The physical properties of the asphalt binders were tested after adding asphalt regenerating agent and the results are shown in Figure 12, Figure 13 and Figure 14.

The results showed that the castor oil-based regeneration agent could restore the physical properties of the three aged asphalt binders. For actual aged asphalt, its penetration, softening point, and ductility could be recovered to 83 dmm, 52.3 °C, and exceeding 100 cm, respectively. For laboratory-aged asphalt, the physical properties of the aged asphalt binders when adding castor oil-based regeneration agent were even better than the original asphalt binder. Therefore, an accidental phenomenon could be ruled out and it was assumed that the castor oil-based asphalt regeneration agent can be applied to most aged asphalt binders.

**Figure 15:**
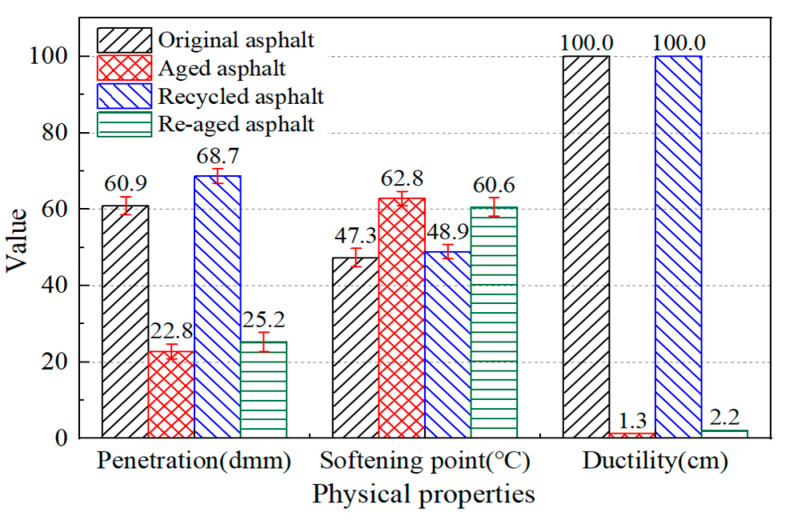
Physical properties of re-aged asphalt and control group asphalt.

Due to the high cost of asphalt pavement renovation, it is necessary to ensure the anti-aging performance of regeneration asphalt. The JL regeneration asphalt was re-aged to study the anti-aging performance of the regeneration agent. The physical properties of the re-aged asphalt binder were tested and the results obtained are shown in Figure 15. Under the same aging conditions, the physical properties were better than those of the JL aged asphalt. This showed that the anti-aging performance of the regeneration asphalt is better than that of the original asphalt from the repeated aging aspect.

**Figure 16:**
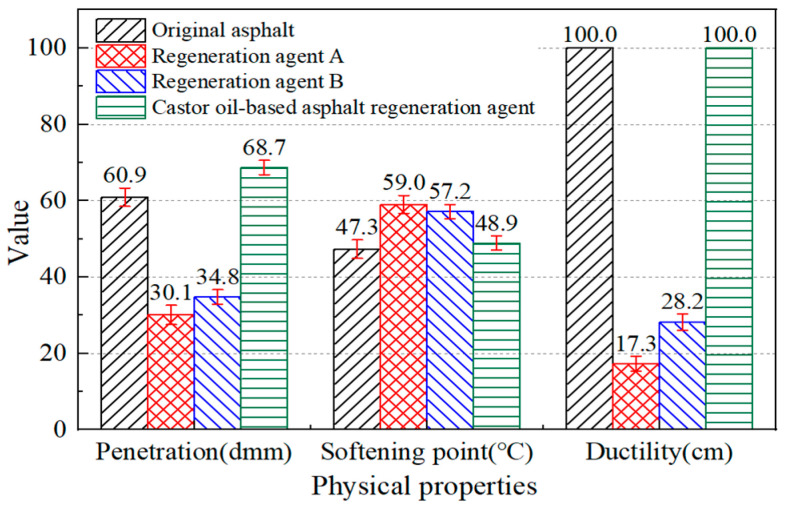
Physical properties of aged asphalt mixed with different asphalt regeneration agents.

Through the above tests, it can be concluded that the castor oil-based asphalt regeneration agent can effectively regenerate aged asphalt and cause its physical properties to return to the level of the original asphalt. In order to compare the regeneration effect of the castor oil-based asphalt regeneration agent, two different kinds of traditional mineral oil-based asphalt regeneration agents, including Regeneration Agent A and Regeneration Agent B, were selected to regenerate aged asphalt by adding 5% of each of them, respectively. Figure 16 shows that all three asphalt reclaiming agents can restore the physical properties of aged asphalt. Compared with the castor oil-based regeneration agent, the Regeneration Agents A and B showed a worse effect on the regeneration of aged asphalt. The softening point was restored to 59.0 °C and 57.2 °C, respectively, and the penetration and ductility did not meet the requirements of the specification. The physical properties of the regeneration asphalt after adding the 5% castor oil-based asphalt regeneration agent were all better than those of the original asphalt. It can be concluded that the developed agent had a better regeneration effect than the traditional mineral oil-based asphalt regeneration agents.

#### 3.3.2. Effect of Regeneration Agent on Pavement Performance of RAP Mixture

The above results indicated that the castor oil-based asphalt regeneration agent had superior improvement effects on the physical properties of aged asphalt binders. Since the effect of regeneration agent on aged asphalt binders and on asphalt mixtures is quite different, it was necessary to add castor oil-based regeneration agent in asphalt mixtures to verify its regeneration performance. In this study, 20%, 40%, 60%, and 80% RAP were used to replace the original aggregate and asphalt binder. The following experiments were carried out on asphalt mixtures with different RAP content.

(1) Moisture Susceptibility Performance

**Figure 17:**
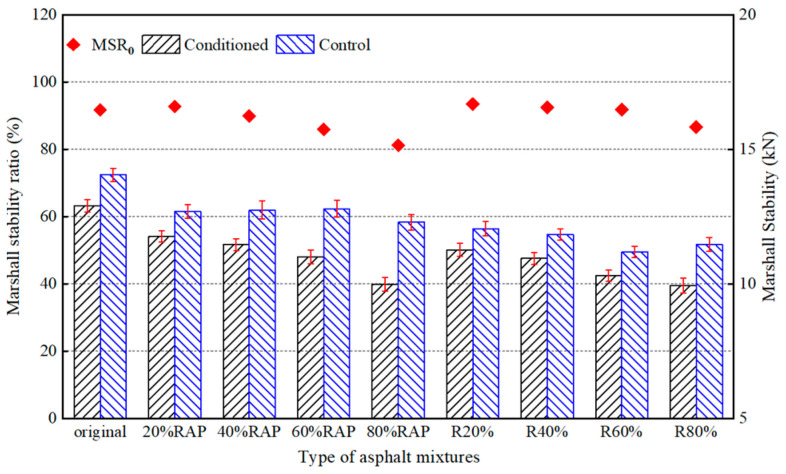
Marshall stability test results of asphalt mixtures.

Figure 17 shows the Marshall stability results of the original asphalt mixture, the RAP asphalt mixtures (20%RAP, 40%RAP, 60%RAP, 80%RAP), and the recycled asphalt mixtures (R20%, R40%, R60%, R80%). Based on the results in Section 3.2, the amount of regeneration agent was determined as 5% by weight of the aged asphalt binder in the recycled asphalt mixture. The Marshall stability ratios of the asphalt mixtures with 20%, 40%, 60%, and 80% RAP were 92.8%, 90.0%, 86.0%, and 81.3%, respectively. The Marshall stability ratio gradually decreased with the increase in RAP content in the RAP asphalt mixtures. The Chinese specification requires that the Marshall stability ratio of hot mix asphalt be greater than 80%. Although the Marshall stability ratios of RAP asphalt mixtures which added 40%, 60%, and 80% RAP were greater than 80%, these were still lower than that of the original asphalt mixture which was 91.8%. This implies that the addition of RAP will make the asphalt mixture more susceptible to moisture effects and reduce the strength of the asphalt mixture. With the addition of the asphalt regeneration agent, the Marshall stability ratio of the recycled asphalt mixture was up about 6% and even higher than that of the original asphalt mixture. It can be concluded that the castor oil-based regeneration agent improved the resistance of RAP asphalt mixtures to moisture based on the results obtained from the Marshall stability test.

**Figure 18:**
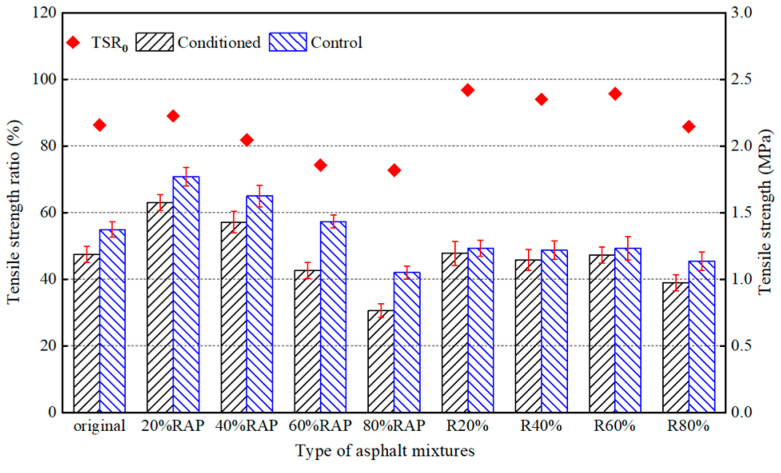
Freeze–thaw split test results of asphalt mixtures.

In addition, we carried out the freeze–thaw split test to evaluate the moisture susceptibility of the asphalt mixtures and the results are shown in Figure 18. The tensile strength ratios of asphalt mixtures decreased with the increase in RAP content. And the TSR values of the 60% and 80% RAP samples were 74.3% and 72.8%, which were lower than the performance requirement of 75% for hot mix asphalt according to the Chinese specification of JTG F40-2004. However, the TSR values of the regenerated samples were all higher than 80% and met the specification. This confirmed the effectiveness of the asphalt regeneration agent on moisture susceptibility.

It is worth noting that the MSR and TSR values of the 20% RAP asphalt mixture were increased compared with those of the original asphalt mixture. The reason might be that the aggregate of the original asphalt mixture was acidic gneiss and the RAP aggregate was alkaline limestone, but the asphalt was a weak acid material. Although aged asphalt in RAP has poor adhesion to aggregates, exposed limestone surfaces during pavement milling have better adhesion to new asphalt than gneiss. The combination effect improved the water stability of the asphalt mixture. The content of new asphalt decreased with the increase in RAP content and consequently the new asphalt adhering to limestone decreased. When the content of RAP was beyond 20%, the negative effect of the RAP content played a dominant role and hence the water stability of asphalt mixture was degraded.

(2) High-Temperature Performance

**Figure 19:**
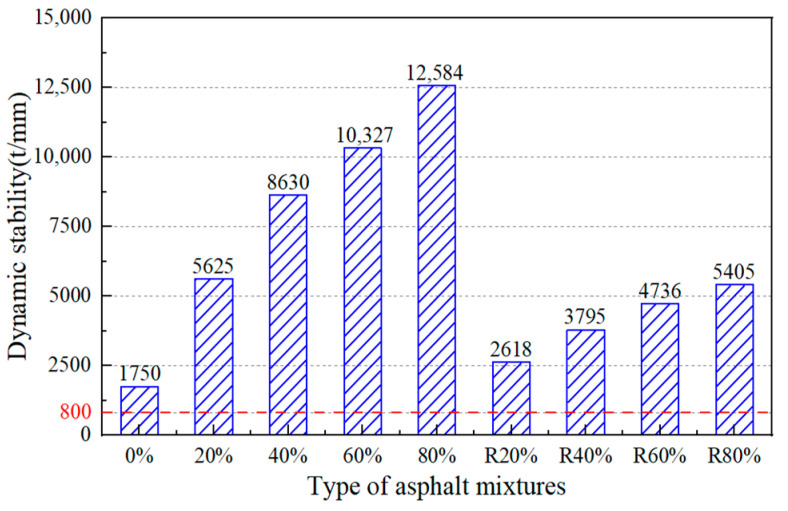
Wheel tracking test results of asphalt mixtures.

Figure 19 shows the wheel tracking test results of the original asphalt mixtures, the RAP asphalt mixtures (20%RAP, 40%RAP, 60%RAP, 80%RAP), and the regenerated asphalt mixtures (R20%, R40%, R60%, R80%). The dynamic stability of the original asphalt mixture and that of the 80% RAP asphalt mixture were the minimum and maximum, respectively. The dynamic stability of the asphalt mixture with 80% RAP content was almost six times higher than that of the original asphalt mixture. Due to the aging effect, the asphalt mixtures with higher RAP content showed greater resistance to permanent deformation under the same repeated loading. Since the regeneration agent softens the RAP in the asphalt mixture, the dynamic stability of the regenerated asphalt mixtures decreased. It can be concluded that the softening effect of the castor oil-based regeneration agent degrades the high-temperature stability of the asphalt mixture, which was in accordance with the results of the study of asphalt.

(3) Low-Temperature Performance

**Figure 20:**
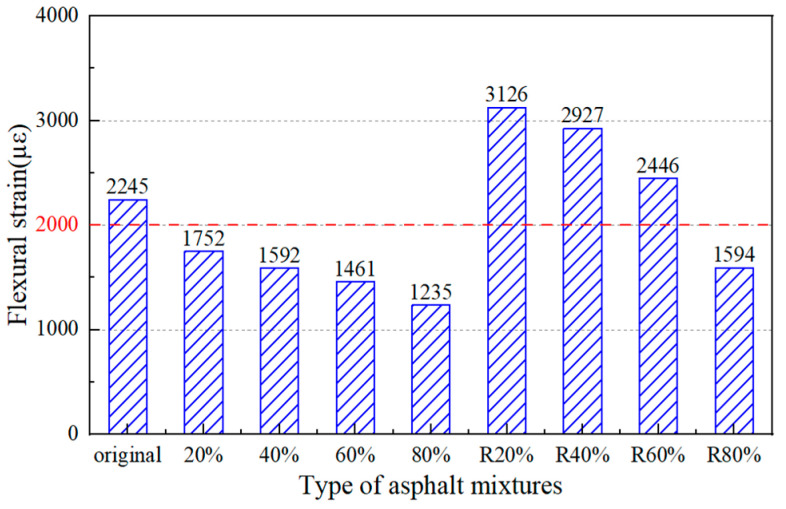
Three-point bending test results of asphalt mixtures.

Figure 20 illustrates the flexural strain of asphalt mixtures with different RAP content and recycled asphalt mixtures. With the increase in RAP content, the flexural strain of the RAP asphalt mixtures decreased gradually and the flexural strain of none of them met the Chinese standard of 2000 µε. The maximum bending strain of the RAP asphalt mixtures was 1752 µε and 1235 µε, respectively, when the content of RAP was 20% and 80%, which was decreased by 21.9% and 44.9% compared with the original asphalt mixture. The reason was that the RAP material degraded the low-temperature performance of the asphalt mixture and increased the risk of cracking of the asphalt pavement. Compared with the RAP asphalt mixtures, the flexural strain of recycled asphalt mixtures was increased due to the effect of the regeneration agent. It can be concluded that the castor oil-based regeneration agent can improve the low-temperature performance of asphalt mixtures.

The recovery rate of the regenerated mixture was 78% when the RAP content was 20%. However, the recovery rate of the regenerated mixture decreased to 29% when the RAP content was 80%. And the flexural strain could not meet the standard requirements in China. Therefore, the RAP content could be determined as 60% according to the low-temperature performance of the asphalt mixtures.

(4) Fatigue Performance

**Figure 21:**
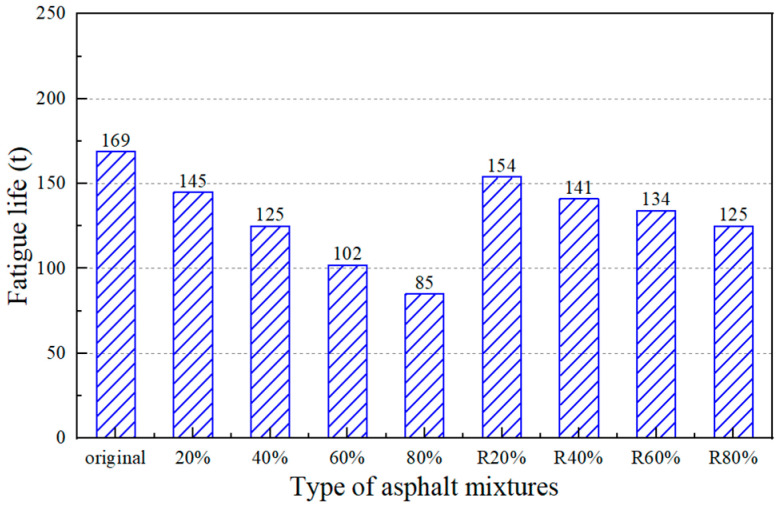
Fatigue life of asphalt mixtures.

Figure 21 illustrates the fatigue lives with different RAP content and recycled asphalt mixtures. The fatigue life of the asphalt mixture decreases with the RAP materials. The fatigue life decreased by 50% when the RAP content was 80%. The lower the fatigue life is, the easier it is for a crack to occur under repeated loading. By comparing the fatigue lives of RAP asphalt mixtures and recycled asphalt mixtures with the same content, it was seen that the fatigue lives of asphalt mixtures were improved after adding a regeneration agent. The fatigue life of the asphalt mixture with 80% RAP recovered by 47.5%. The results showed that the recovery effect of the castor oil-based regeneration agent on fatigue life was strong. Although the performance of the original asphalt mixture cannot be achieved, it can restore the fatigue life of an RAP asphalt mixture to a similar level.

(5) Anti-aging Performance

When the content of RAP was 60%, the pavement performance of regenerated asphalt mixtures met the Chinese standard. Therefore, asphalt mixtures containing 60% RAP were prepared to investigate the anti-aging performance. First, the recycled asphalt mixture was heated in an oven at 135 °C ± 3 °C for 4 h ± 5 min (short-term aging), and then in an oven at 85 °C ± 3 °C for 120 h ± 0.5 h (long-term aging). After aging the recycled asphalt mixture in the laboratory, the pavement performance of the original asphalt mixture (0% RAP), the aged asphalt mixture (60% RAP), the recycled asphalt mixture (R60% RAP), and the re-aged asphalt mixture (2–60% RAP) was tested to study the anti-aging performance of recycled asphalt mixtures.

**Figure 22:**
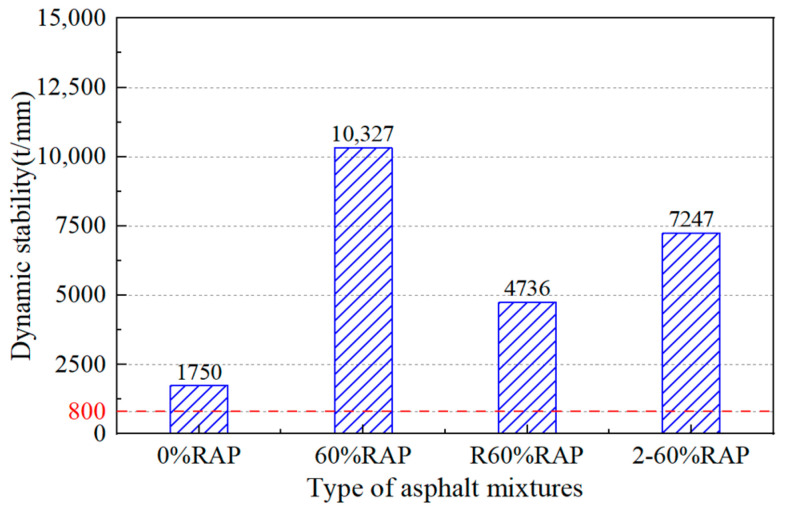
Wheel tracking test results of re-aged asphalt mixtures.

Figure 22 shows the dynamic stability of the asphalt mixtures measured by the rutting test. The dynamic stability of the re-aged asphalt mixture was 7247 t/mm, which was 53% higher than that of the recycled asphalt mixture and four times that of the original asphalt mixture, while the result of the aged asphalt mixture was six times that of the original asphalt mixture. A greater increase in the dynamic stability of an asphalt mixture means the aging degree of asphalt is deeper. This shows that the anti-aging performance of the recycled asphalt mixture is strong.

**Figure 23:**
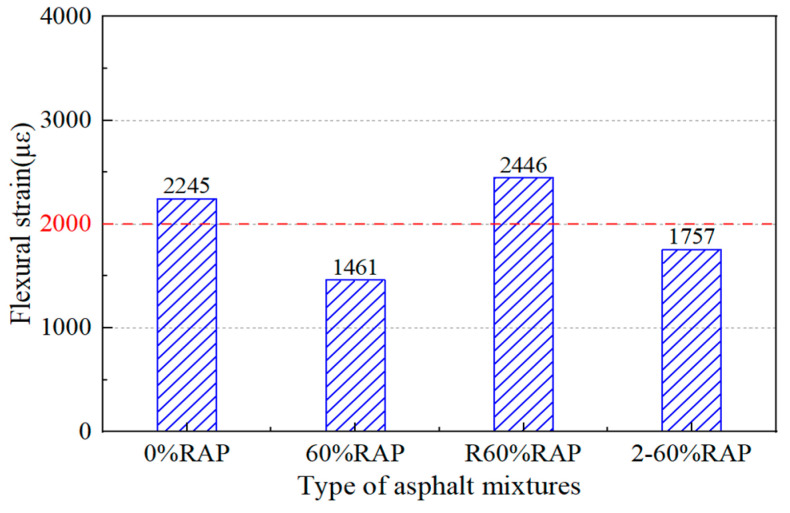
Three-point bending test results of re-aged asphalt mixtures.

As shown in Figure 23, the maximum flexural tensile strain of the re-aged asphalt mixture and the aged asphalt mixture was 1757 µε and 1461 µε, respectively, which were 22% and 35% lower than that of the original asphalt mixture. Since the maximum flexural strain of the re-aged asphalt mixture was greater than that of the aged asphalt mixture, this indicates that the anti-aging performance of the recycled asphalt mixture was excellent as well.

**Figure 24:**
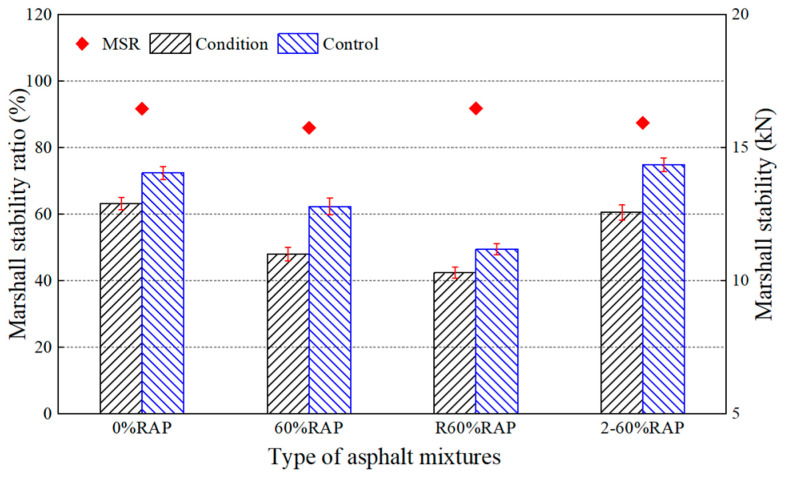
Marshall stability test results of re-aged asphalt mixtures.

**Figure 25:**
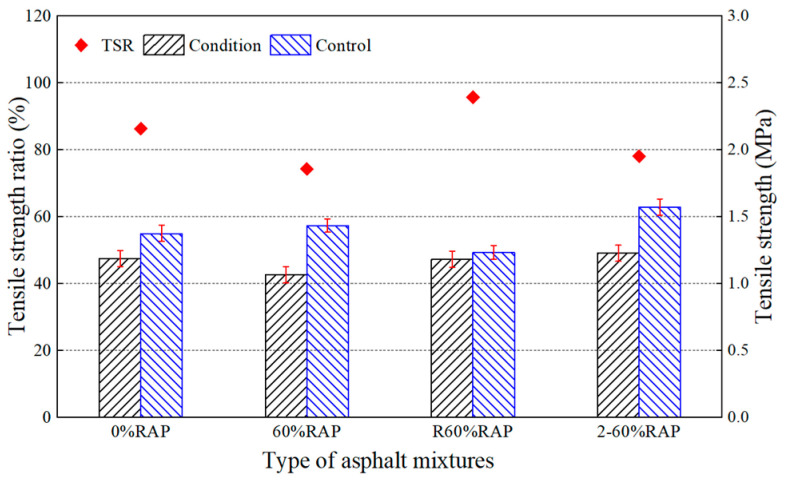
Freeze–thaw split test results of re-aged asphalt mixtures.

Figure 24 and Figure 25 show that the Marshall stability ratio and the tensile strength ratio of the re-aged asphalt mixture were 87.5% and 78.1%, respectively, which were higher than those of the aged asphalt mixture. This implies that the water stability of the re-aged asphalt mixture was better. It is well-known that the aging effect will reduce the Marshall stability ratio and tensile strength ratio of an asphalt mixture. Compared with the original asphalt mixture, the Marshall stability ratio and tensile strength ratio of the aged asphalt mixture decreased by 6.2% and 12.3%, respectively, while the re-aged asphalt mixture only by 4.7% and 9.6%. All the pavement performance tests showed that the anti-aging performance of the recycled asphalt mixture is desirable. Therefore, the recycled asphalt mixture can be expected to be used in an asphalt pavement for a long time.

## 4. Conclusions

In this study, a new castor oil-based asphalt regeneration agent was developed and its effects on aged asphalt binders and RAP materials were comparatively investigated by a series of laboratory tests. According to the test results, conclusions could be drawn as follows.

1. The castor oil-based asphalt regeneration agent was developed by a series of tests on laboratory-aged asphalt. It consists of castor oil and dibutyl phthalate at a weight ratio of 1:4. The optimum content of the regeneration agent was 5% by the weight of the aged asphalt binder in this study.
2. The regeneration effectiveness of the castor oil-based regeneration agent was tested with three laboratory-aged asphalt binders and an actual aged asphalt binder, and the anti-aging performance of the recycled asphalt was good. The regeneration effect of the castor oil-based asphalt regeneration agent was better than that of the two traditional mineral oil-based asphalt regeneration agents.
3. The castor oil-based regeneration agent can effectively restore the pavement performance of an RAP asphalt mixture, especially the low-temperature anti-cracking performance. The content of the RAP can reach 60% by the weight of the asphalt mixture with the castor oil-based regeneration agent. The anti-aging performance of the recycled asphalt mixture was better than that of the original asphalt mixture, so it can be used in an asphalt pavement for a long time.

Overall, this paper provides a reference for the material design and performance evaluation of a castor oil-based regeneration agent. However, there are still some problems that can be explored in future research, including the following aspects:

(1) The universality of the castor oil-based asphalt regeneration agent. The asphalt binders and RAP materials used in this study were limited. In future research, asphalts from different sources and RAP materials of different gradation and different years could be added to verify the regeneration performance of the castor oil-based asphalt regeneration agent.
(2) The regeneration mechanism of the castor oil-based asphalt regeneration agent. Combined with advanced material testing technology, determining the effect mechanism of the castor oil-based asphalt regeneration agent on aged asphalt in RAP, including composition adjustment, interface fusion, etc., would provide a theoretical basis and technical support for the engineering application of the castor oil-based asphalt regeneration agent.
(3) It is strongly recommended to pave a pavement test section to investigate the pavement performance and durability of the RAP asphalt mixture with the castor oil-based regeneration agent.

## Figures and Tables

**Table 1:** Physical properties of original asphalts.

Physical Properties	JL AH-70	SL AH-70	Shell AH-70
Penetration (dmm)	60.9	73.1	60.3
Softening point (°C)	47.3	46.6	50.8
Ductility (cm)	>150	>150	>150

**Table 2:** Performance of raw materials for preparing asphalt regeneration agent.

Performance	Castor Oil	Plasticizer ESO	Plasticizer TC	Plasticizer DP
Viscosity (mPa·s)	84	325	32	96
Flash Point (°C)	322	310	368	172
Density (g/mL)	0.963	0.985	1.042	1.045
Toxicity	non-toxic	non-toxic	low toxicity	slightly toxic

**Table 3:** Physical properties of aged asphalt.

Physical Properties	Long-Term Aging			Actual Aged Asphalt
	JL AH-70	SL AH-70	Shell AH-70	
Penetration (dmm)	23.7	29.1	21.7	29.2
Softening point (°C)	63.6	60.7	68.9	64.3
Ductility (cm)	1.0	2.2	0.9	14.2

## Data Availability

All the data in the tests of this study have been listed in the paper.
